# Anticoagulation Therapy by Age and Embolic Risk for Nonvalvular Atrial Fibrillation in Mexico, an Upper-Middle-Income Country: The CARMEN-AF Registry

**DOI:** 10.5334/gh.767

**Published:** 2020-04-10

**Authors:** Manlio F. Márquez, Manuel A. Baños-González, Milton E. Guevara-Valdivia, Jorge Vázquez-Acosta, Manuel O. de los Ríos Ibarra, Julio A. Aguilar-Linares, Marcelo Jiménez-Cruz, Norberto Matadamas-Hernández, Rocío Camacho-Casillas, Reynaldo Magaña-Magaña, Ulises Rojel-Martínez, Marco A. Alcocer-Gamba, Susano Lara-Vaca, Humbert Rodríguez-Reyes, Marco A. Islava-Gálvez, Lidia E. Betancourt-Hernández, Nicolás Reyes-Reyes, Miguel E. Beltrán-Gámez, Carlos Cantú-Brito, Alberto Z. Baños-Velasco, Pedro J. del Rivero Morfin, J. Antonio González-Hermosillo

**Affiliations:** 1 Instituto Nacional de Cardiología Ignacio Chávez, MX; 2 Centro de Investigación y Posgrado, División Académica de Ciencias de la Salud, Universidad Juárez Autónoma de Tabasco (UJAT), MX; 3 Unidad Médica de Alta Especialidad del Hospital de Especialidades “Dr. Antonio Fraga Mouret”, Centro Médico Nacional “La Raza”, Instituto Mexicano del Seguro Social, MX; 4 Hospital Regional de PEMEX Ciudad Madero, MX; 5 SINACOR, Centro para el Desarrollo de la Medicina y de Asistencia Médica Especializada S.C., MX; 6 Hospital Regional No.1 Tijuana, Instituto Mexicano del Seguro Social, MX; 7 Hospital de Cardiología, Centro Médico Nacional “Siglo XXI”, Instituto Mexicano del Seguro Social, MX; 8 Hospital General de Acapulco, MX; 9 Unidad Médica de Alta Especialidad No. 71, Instituto Mexicano del Seguro Social, MX; 10 Hospital General de Uruapan “Dr. Pedro Daniel Martínez”, MX; 11 Hospital General del Sur de Puebla “Eduardo Vázquez Navarro”, MX; 12 Instituto de Corazón de Querétaro, MX; 13 Hospital Ángeles León, MX; 14 Sociedad Cardiovascular y Arritmias, MX; 15 Instituto Mexicano de Trasplantes, MX; 16 Unidad Médica de Alta Especialidad No. 14, Instituto Mexicano del Seguro Social, MX; 17 Hospital Ángeles Puebla, MX; 18 Hospital Ángeles Tijuana, MX; 19 Departamento de Neurología, Instituto Nacional de Ciencias Médicas y Nutrición “Salvador Zubirán”, MX; 20 Hospital Ángeles Culiacán, MX

**Keywords:** Atrial fibrillation, embolic risk, anticoagulation therapy, antithrombotic treatment, vitamin K antagonist, direct oral anticoagulant, Mexico

## Abstract

**Background:**

Documenting the patterns of oral anticoagulation therapy (OAT) is essential to prevent thromboembolic complications of nonvalvular atrial fibrillation (NVAF).

**Objective:**

To report the patterns of OAT according to age and thromboembolic risk in patients included in CARMEN-AF, a nationwide registry of NVAF in Mexico, an upper middle-income country.

**Material and methods:**

There were 1,423 consecutive patients ≥18 years old and with at least one thromboembolic risk factor enrolled in the CARMEN-AF Registry at their regular clinical visit during a three-year period. They were analyzed according to 1) age, 2) AF type, and 3) CHA_2_DS_2_-VASc score.

**Results:**

Overall, 16.4% of patients did not receive antithrombotic treatment, 19.4% received antiplatelet drugs (APD), 29.2% vitamin K antagonists (VKA), and 34.6% direct oral anticoagulants (DOAC). With increasing age, the proportion of subjects treated with VKA decreased significantly from 36.2% in subjects <65 years to 22.5% in those ≥75 years old (*P* <0.0001). Concomitantly, an increase in both APD and no antithrombotic treatment was observed with increasing age. DOAC were prescribed equally among all age groups (34.2% in <65, 36.0% in 65–74, and 33.9% in ≥75). According to the type of AF, VKA use was more common in patients with permanent AF (32.7%). A lower use of DOAC was observed in high thromboembolic risk subjects (33.6% in CHA_2_DS_2_-VASc ≥2) compared with the moderate risk group (41% in CHA_2_DS_2_-VASc = 1).

**Conclusions:**

VKA use for NVAF in Mexico decreased in relation to increasing age. The proportion of DOAC therapy was the same in all age groups. Nevertheless, elderly patients with high thromboembolic risk received a suboptimal thromboprophylaxis. These data could help to improve gaps in the implementation of global guidelines.

**Clinical trial registration:**

http://www.clinicaltrials.gov. Unique identifier: NCT02334852.

**Highlights:**

- CARMEN-AF is a nationwide multi-centric registry seeking to bridge the data gap on anticoagulation therapy for NVAF in Mexico.
- Elderly patients are more prone to receive suboptimal OAT for NVAF.
- DOAC were less frequently used in high thromboembolic risk patients (CHA_2_DS_2_-VASc ≥2).

## Introduction

Oral anticoagulation therapy (OAT) is the cornerstone of stroke prevention in nonvalvular atrial fibrillation (NVAF) [1]. Current registries of anticoagulation therapy (ACT) for NVAF are a priority in high-income countries [2,3]. However, in upper middle-income countries like Mexico, there is scarce epidemiological data about OAT for NVAF [4,5].

CARMEN-AF (Registry of Atrial Fibrillation and Embolic Risk in Mexico) is a nationwide, industry-independent registry, developed as a means to bridge the information gap about OAT for NVAF in Mexico [6]. Herein, we report the results of the CARMEN-AF Registry, analyzed by age, AF type, and thromboembolic risk.

## Material and Methods

CARMEN-AF is an ongoing, observational, longitudinal, multi-center, nationwide registry of OAT in NVAF in Mexico. A full list of the investigators of the Registry could be consulted in the Appendix A of this article. The protocol has already been published [6]. An English version of the Registry of Atrial Fibrillation and Embolic Risk in Mexico (CARMEN-AF) could be consulted in Supplementary Material. Mexico’s economic status as an upper-middle income country is based on the 2013 World Bank Classification according to gross national income per capita [7]. A total of 1,423 consecutive patients were enrolled in a three-year period (September 19, 2014 – December 18, 2017).

### Study population

Eligible patients were at least 18 years old, with one or more risk factors for thromboembolism evaluated by CHA_2_DS_2_-VASc score and diagnosed with NVAF at least 6 months prior to their inclusion. Patients were included independent of anti-thrombotic therapy (ATT) (antiplatelet drugs [APD], vitamin K antagonists [VKA] or direct oral anticoagulants [DOAC] available in Mexico [dabigatran, rivaroxaban, and apixaban]). We used cross-sectional data obtained at patient recruitment. Physicians performed data collection at regular clinic visits using a paper-based case report form, with subsequent capture in an electronic case report form for data storage.

A table specifying the centers that participated, grouped by state (geographical distribution), and details about the type of clinic (public/private, second/third level), and the type of practice (the specialty of the center coordinator) can be consulted in the supplementary material. It also includes the type of residence of the patients (urban or rural). All patients were treated in urban centers (mostly by cardiologists), representing 29 states of the Mexican Republic. All patients were assessed at an initial clinical visit with a complete medical history and physical examination (Table 1).

**Table 1:** Geographical distribution (Mexican states) of subjects included in CARMEN-AF and other related variables.

Center	State	Level of care	Type	Specialty of the coordinator	Type of residency of the patients (Urban, rural, both)

Sociedad Cardiovascular de Aguascalientes	Aguascalientes	Third	Private	Electrophysiologist	Urban
Hospital Hidalgo	Aguascalientes	Third	Public	Cardiologist	Both
Hospital Regional No1 IMSS, Tijuana	Baja California	Second	Public	Cardiologist	Both
Hospital Angeles Tijuana	Baja California	Third	Private	Cardiologist/Internist	Urban
Plaza Medical	Baja California	Third	Private	Cardiologist	Urban
Hospital General ISSSTE, La Paz	Baja California sur	Third	Public	Cardiologist/Internist	Urban
Hospital GE ‘Dr Javier Buenfil Osorio’ INDESALUD	Campeche	Third	Public	Cardiologist	Both
Instituto Nacional de Cardiologist ‘Ignacio Chavez’	Mexico City	Third	Public	Electrophysiologist	Both
Hospital de Especialidades, Centro Medico Nacional ‘La Raza’ IMSS	Mexico City	Third	Public	Electrophysiologist	Both
Hospital de Cardiologist del Centro Medico Nacional ‘Siglo XXI’ IMSS	Mexico City	Third	Public	Electrophysiologist	Both
Hospital General de Mexico	Mexico City	Third	Public	Electrophysiologist	Urban
Instituto Nacional de Ciencias Medicas y Nutricion ‘Salvador Zubiran’	Mexico City	Third	Public	Neurologist	Urban
CIMA Chihuahua	Chihuahua	Third	Private	Cardiologist	Urban
Unidad Medica de Alta Especialidad No71 IMSS, Coahuila	Coahuila	Third	Public	Cardiologist	Urban
Hospital General de Zona No 10, Manzanillo	Colima	Second	Public	Cardiologist/Internist	Urban
Hospital General de Zona No 1 IMSS, Durango	Durango	Second	Public	Cardiologist/Internist	Both
ISSEMYM Toluca	Estado de Mexico	Third	Public	Electrophysiologist	Urban
Hospital Angeles Leon	Guanajuato	Third	Private	Electrophysiologist	Both
Hospital General de Acapulco	Guerrero	Third	Public	Cardiologist/Internist	Both
Hospital General de Pachuca	Hidalgo	Third	Public	Cardiologist/Internist	Both
Hospital Civil de Guadalajara	Jalisco	Third	Public	Cardiologist/Internist	Urban
Hospital General de Uruapan	Michoacan	Second	Public	Cardiologist	Both
Instituto Nacional de Trasplantes	Morelos	Second	Private	Cardiologist/Internist	Urban
Instituto de Cardiologist y Medicina Vascular Hospital Zambrano, Tec Salud	Nuevo Leon	Third	Private	Electrophysiologist	Urban
Hospital Regional de Alta Especialidad de Oaxaca	Oaxaca	Third	Public	Cardiologist. Internist	Urban
Clinica Molina	Oaxaca	Segunda	Public	Cardiologist	Urban
Hospital General del Sur de Puebla	Puebla	Third	Private	Electrophysiologist	Urban
Hospital Angeles de Puebla	Puebla	Third	Private	Electrophysiologist	Urban
Instituto del Corazon Queretaro	Queretaro	Third	Private	Internist	Both
Hospital General de Zona No3 IMSS	Quintana Roo	Second	Public	Cardiologist	Both
Hospital Central ‘Dr Ignacio Morones Prieto’	San Luis Potosi	Third	Public	Cardiologist	Both
Torre Medica Olivos	San Luis Potosi	Primer	Private	Cardiologist	Urban
Hospital Civil de Culiacan	Sinaloa		Public	Cardiologist	Both
Angeles Culiacan	Sinaloa	Third	Private	Cardiologist/Internist	Urban
Hospital General de Culiacan	Sinaloa	Second	Public	Cardiologist/Internist	Both
Centro Medico Nacional del Noroeste IMSS	Sonora	Third	Public	Cardiologist	Both
Hospital Regional de Alta Especialidad ‘Juan Graham Casasus’	Tabasco	Third	Public	Cardiologist/Internist	Both
Hospital Regional de PEMEX Ciudad Madero	Tamaulipas	Third	Public	Cardiologist	Both
Unidad Medica de Alta Especialidad No 14 IMSS	Veracruz	Third	Public	Cardiologist	Both
Star Medica Merida	Yucatan	Third	Private	Cardiologist	Urban
Hospital ‘San Agustin’	Zacatecas	Third	Private	Cardiologist	Both

### Statistical methodology

Data was analyzed using SPSS v. 22.0. Demographic differences among continuous variables with normal distribution were examined using Student’s t-test; Wilcoxon signed-rank test was used when variables failed normality test. Categorical variables were analyzed using Chi-square test, either Fisher’s exact test or Yates’s correction for continuity. A 2-tail test with a *P* value <0.05 was considered statistically significant.

### Informed consent

Informed consent was obtained from each patient and the study protocol conforms to the ethical guidelines of the 1975 Declaration of Helsinki as reflected in a priori approval by the institution’s human research committee.

## Results

A total of 1,423 consecutive patients were enrolled in a three-year period (September 19, 2014–December 18, 2017). Mean age of participants was 69 ± 13 years old. 731 (51.4%) were male. Complete demographic characteristics are shown in Table 2.

**Table 2:** Demographic characteristics.

Demographic characteristics				

	All(n = 1,423)	Male(n = 731)	Female(n = 692)	P*

Gender (%)		51.4	48.6	ns
Age, years ± SD	69 ± 13	68 ± 13	70 ± 12	=0.002
Weight, kg ± SD	75 ± 16	80 ± 15	69 ± 14	<0.0001
Body Mass Index, kg/m^2^ ± SD	28.5 ± 5.0	28.4 ± 4.6	28.7 ± 5.4	ns

* *P* value was obtained comparing Gender groups using Chi-square test and Student’s t-test.

NVAF was paroxysmal in 37.3% of the cases, persistent in 22.1%, permanent in 40.6%; AF was asymptomatic in 59.4% of patients. The most prevalent comorbidities (Table 3) in patients with NVAF were hypertension (72.5%), diabetes (28.4%), heart failure (23.6%) and smoking (16.4%).

**Table 3:** Comorbidities of total population.

Comorbidities				

(%)	All (n = 1,423)	Male (n = 731)	Female (n = 692)	P*

Hypertension	72.5	71.3	73.8	ns
Diabetes	28.4	31.3	25.3	=0.007
Heart failure	23.6	25.3	21.8	ns
Smoking	16.4	23.9	8.5	<0.0001
Alcoholism	9.2	17.1	0.9	<0.0001
Nonischemic cardiomyopathy**	8.9	10.3	7.5	=0.042
Coronary Artery Disease	7.1	9.7	4.3	<0.0001
Obstructive sleep apnea	3.9	5.2	2.6	=0.008
Peripheral artery disease	1.8	1.0	2.7	=0.010

* *P* value was obtained comparing Gender groups using Chi-square test and Student’s t-test. ** Hipertensive, Idiopathic, and restrictive.

Related to ATT, 16.6% of patients were not receiving any; 19.4% were receiving APD, and 63.9% of the patients were receiving oral anticoagulants (VKA = 416, 29.2%; DOAC = 493, 34.6%). OAT was either monotherapy (56.9%) or combined with one or two APD (7.2%) (Table 4).

**Table 4:** Antithrombotic therapy according to AF type.

Antithrombotic therapy according to AF type				

(%) *P* = 0.037*	All (n = 1,423)	Paroxysmal (n = 531)	Persistent (n = 314)	Permanent (n = 578)

Without treatment	16.6	17.5	15.9	16.1
Antiplatelet	19.4	22.4	19.7	16.4
Anticoagulant	56.8	53.7	54.1	61.1
Anticoagulant + Antiplatelet	5.7	4.9	8.9	4.7
Triple therapy	1.5	1.5	1.3	1.7

* *P* value was obtained comparing Gender groups using Chi-square test and Student’s t-test.

### Antithrombotic therapy by AF type

**Figure 1:**
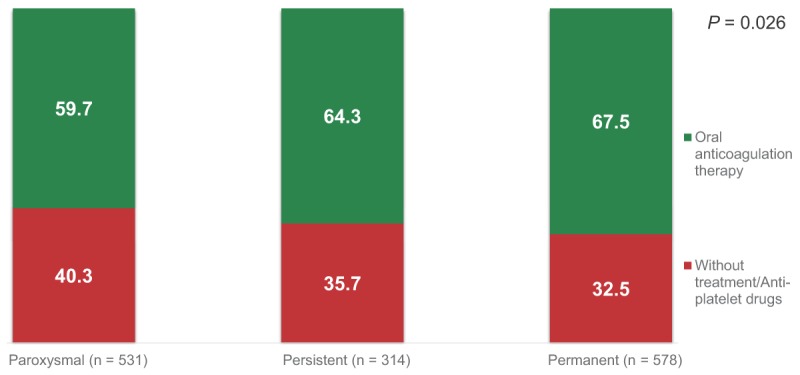
Antithrombotic therapy by AF type (%). * *P* value was obtained comparing AF type groups vs Treatment groups using Chi-square test.

In accordance to AF type (paroxysmal, persistent, and permanent), suboptimal ATT (no ATT or just APD treatment) was observed in 40.3% of patients in the paroxysmal group, 35.7% in the persistent group, and 32.5% in the permanent group. There was a statistically significant difference between treatments when the groups were compared. (*P* = 0. 026) (Figure 1).

### Antithrombotic therapy by thromboembolic risk

**Figure 2:**
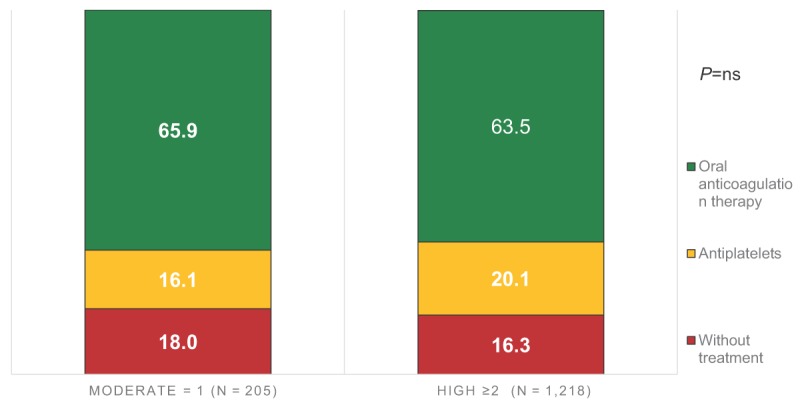
Antithrombotic therapy according to CHA_2_DS_2_-VASc risk (%). * *P* value was obtained comparing Treatment groups vs CHA_2_DS_2_-VASc groups using Chi-square test.

The thromboembolic risk was assessed using CHA_2_DS_2_-VASc score (moderate risk = 1 point; high risk ≥2 points). No significant difference was observed on ATT between moderate and high thromboembolic risk subjects. Interestingly, inadequate ATT was observed in 36.4% of high thromboembolic risk patients: 20.1% were treated with APD and 16.3% did not receive any treatment (Figure 2).

### Antithrombotic therapy by age

**Figure 3:**
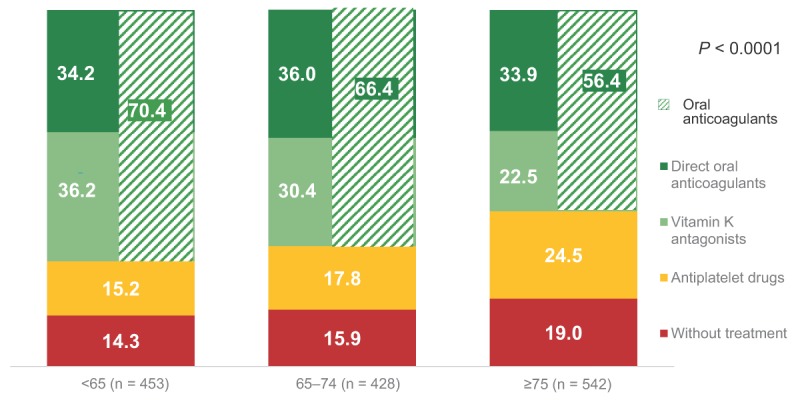
Antithrombotic therapy according to age (%). * *P* value was obtained comparing Treatment groups vs Age groups using Chi-square test.

As the CHA_2_DS_2_-VASc score classifies thromboembolic risk according to age, patients were divided into three groups (Figure 3). In the group of patients younger than 65 years old, 70.4% received OAT (DOAC 34.2%, VKA 36.2%), 15.2% received APD and 14.3% did not receive ATT. In the 65–74 years old group, 66.4% of patients received OAT (DOAC 36.0%, AVKs 30.4%), 17.8% received APD, and 15.9% did not receive ATT. In the group of patients’ ≥75 years old, 56.4% received OAT (DOAC 33.9.0%, VKA 22.5%), 24.5% were on APD, and 19.0% received no treatment.

### Bleeding risk

There was a significant difference in HAS-BLED score between men and women; it was higher in men (1.82 ± 1.0 vs 1.71 ± 0.9; *P* = 0.032). According to bleeding risk, more women had a low risk of bleeding than men (9.7% vs 6.3%, *P* = 0.012), while more men had a high risk (20.8% vs 16.8%, *P* = 0.030) [19].

Of the studied population, 9.5% of women and 19.2% men at high risk of bleeding (HAS-BLED score = ≥3) did not receive any antithrombotic therapy (*P* = 0.021).

## Discussion

Patients with AF have 5 times greater risk of stroke than the general population [8]. There is significant evidence that the use of oral anticoagulants, both VKA and DOAC, reduce the risk of stroke in patients with NVAF [9,10,11,12]. Thromboembolic risk assessment should be performed at diagnosis using CHA_2_DS_2_-VASc score, and OAT should be started in patients with moderate and high risk of thrombosis (score ≥ 2) [1].

Clinical registries are proven to evaluate the correct application of treatment guidelines.

CARMEN-AF was designed to find out current information about the status of OAT for NVAF in Mexico. The main finding of this registry was that despite most of the patients in our cohort (85.6%) were classified as high thromboembolic risk according to CHA_2_DS_2_-VASc score (≥2), 35.8% of the total population diagnosed with NVAF were not receiving OAT or only received APD, a suboptimal therapy.

It was also found a trend in the use of OAT according to age. In Mexico, OAT is less commonly prescribed for elderly patients, despite that age is a well-known risk factor for thromboembolism; this conduct may be related to the fear of hemorrhagic complications in this group of patients according to HAS-BLED score [13]. Elderly patients who did receive OAT were more likely to be prescribed with a DOAC instead of VKA, probably due to better adherence and ease of use of DOAC [14].

Data collected by CARMEN-AF shows similarities with other large-scale NVAF registries. In Mexico, an upper middle-income country, hypertension remains the main comorbidity, just as in GLORIA-AF and GARFIELD registries. Also, the proportion of participants with high thromboembolic risk as assessed by CHA_2_DS_2_-VASc scores was very similar among the three registries (GLORIA-AF 86.1%; GARFIELD 84.3%; CARMEN-AF 85.6%). Finally, in Mexico DOAC are preferred as OAT in contrast with some other countries in Latin America, in which VKA remains the therapy of choice for prevention of thromboembolic complications in NVAF [15,16,17].

In order to understand differences among registries, we must remember that CARMEN-AF had a greater proportion of permanent NVAF (40.6%), while GLORIA-AF reported only 11.1% and GARFIELD 13.1% (*P* < 0.0001). Also, the amount of patients left untreated in CARMEN-AF (16.6%) is greater than GARFIELD (12.3%) and GLORIA-AF (7.8%) global cohorts, as well as GLORIA-AF Latin America cohort (4.2%) [15, 16, 18]. Significant differences in treatment were observed between the three registries (*P* < 0.0001).

## Limitations

CARMEN-AF was based on the prescription of antithrombotic therapy by different specialists; therefore, our data may not apply to other healthcare givers.

According to protocol, this survey recruited only patients with CHA2DS2-VASc≥ 1. Thus, no data on patients with score zero (low risk of stroke) were available.

Both patients and physicians knew they were participants of a registry; this might have led to higher overall anticoagulation rates compared with general population.

Unfortunately, the socioeconomic status as a variable is not available for the entire study.

## Conclusions

CARMEN-AF demonstrated a suboptimal thromboprophylaxis in NVAF in Mexico, an upper-middle income country, accounting for relevant differences with respect to high-income countries. Identification of gaps in the implementation of global guidelines between countries is the first step towards the objectives of the World Heart Federation Roadmap for NVAF.

## Additional File

The additional files for this article can be found as follows:

10.5334/gh.767.s1 Appendix A. Full list of the investigators and their participating centers of the Registry of Atrial Fibrillation and Embolic Risk in Mexico (CARMEN-AF).

10.5334/gh.767.s2 Supplementary material. A full English version of the design of the Registry of Atrial Fibrillation and Embolic Risk in Mexico (CARMEN-AF), to assist our global audience to a better approaching to the original article published in Spanish in 2016 in Archivos de Cardiología de México.

## Abbreviations

AF = Atrial fibrillation

APD = Antiplatelet drugs

ATT = Antithrombotic therapy

DOAC = Direct oral anticoagulants

NVAF = Nonvalvular atrial fibrillation

OAT = Oral anticoagulation therapy

VKA = Vitamin K antagonists

## Clinical Trial Registration

http://www.clinicaltrials.gov. Unique identifier: NCT02334852.
