# Supplementary material for: Anticoagulation Therapy by Age and Embolic Risk for Nonvalvular Atrial Fibrillation in Mexico, an Upper-Middle-Income Country: The CARMEN-AF Registry
Source: Glob Heart. 2020 Apr 10;15(1):32. doi: 10.5334/gh.767 (PMC7218765; doi:10.5334/gh.767)
Supplement: Appendix A. — Full list of the investigators and their participating centers of the Registry of Atrial Fibrillation and Embolic Risk in Mexico (CARMEN-AF). [file gh-15-1-767-s1.pdf]

APPENDIX A

Investigators of the CARMEN-AF Registry are as follows:

María Sangeado Santos, César González Aguilar; Hospital Regional de Alta Especialidad "Juan Graham Casasús" SSA, Villahermosa, Tabasco. Jorge Gómez Flores; Instituto Nacional de Cardiología “Ignacio Chávez, Ciudad de México. Karim Yarek Juárez Escobar, Hospital de Especialidades del Centro Médico Nacional “La Raza” IMSS, Ciudad de México. Samantha Rodríguez Bastidas, José Raúl de los Ríos Ibarra; Hospital Civil de Culiacán, Sinaloa. Carmen Summerson Lama; Hospital Regional No.1 IMSS, Tijuana, Baja California. Enrique Martínez Flores; Hospital de Cardiología del Centro Médico Nacional “Siglo XXI” IMSS, Ciudad de México. Vanesa Esperanza Matadamas Carmona, Candelaria Mancilla; Hospital General de Acapulco, Guerrero. José Refugio Ramírez González; Unidad Médica de Alta Especialidad No.71 IMSS, Torreón, Coahuila. José Luis Leiva Pons, Jorge Carrillo Calvillo; Hospital Central “Dr. Ignacio Morones Prieto”, San Luis Potosí, San Luis Potosí. Héctor Francisco Fernández Saldaña, Laura Reséndiz Barrón; Instituto del Corazón Querétaro, Querétaro. Edgar Alfredo Rodríguez Salazar; Hospital Ángeles León, Guanajuato. José Salvador Lainez Zelaya; Instituto Nacional de Trasplantes, Cuernavaca, Morelos. Yoloxochitl García Jiménez, Sadoc Marín Rendon; Unidad Médica de Alta Especialidad No. 14 IMSS, Veracruz, Veracruz. Luis Gerardo Molina Fernández de Lara, Carlos Gutiérrez González; Hospital General de México, Ciudad de México. Fernando Flores Silva; Instituto Nacional de Ciencias Médicas y Nutrición “Salvador Zubirán”, Ciudad de México. Gerardo Pozas Garza; Hospital Zambrano Hellion, Monterrey, Nuevo

León. Eddie Alberto Favela Pérez; Star Medica Mérida, Yucatán. Amanda Castelán Ojeda, César Iván Vázquez Serna; Centro Médico Nacional del Noroeste IMSS, Ciudad Obregón, Sonora. Miguel Ángel Negrete Rivera; ISSEMYM Toluca, Estado de México. Alejandro Lechuga Martín del Campo; Hospital General de Pachuca, Hidalgo. Demetrio Kosturakis García; CIMA Chihuahua, Chihuahua. Ramón Miguel Esturau Santaló; Hospital Civil de Guadalajara, Jalisco. José Luis Novelo Del Valle; Hospital GE "Dr. Javier Buenfil Osorio" INDESALUD, Campeche, Campeche. Luis Delgado Leal; Hospital Hidalgo, Aguascalientes, Aguascalientes. Luis Ángel Trujillo Muñoz; Hospital General de Zona No. 10, Manzanillo, Colima. José Fabián Hernández Díaz, Mariano Miguel Guerra, Hospital Regional de Alta Especialidad de Oaxaca, Oaxaca, Oaxaca. Raúl Isaac Márquez; Hospital General de Zona No.3 IMSS, Cancún, Quintana Roo. María Isabel Sánchez Ramírez, Marcos Robledo; Hospital General ISSSTE, La Paz, Baja California Sur. José Manuel Enciso Muñoz; Hospital "San Agustín", Zacatecas, Zacatecas. Juan Carlos Núñez Fragoso, Saúl Flores; Hospital General de Zona No. 1 IMSS, Durango, Durango.
