# Supplementary material for: Anticoagulation Therapy by Age and Embolic Risk for Nonvalvular Atrial Fibrillation in Mexico, an Upper-Middle-Income Country: The CARMEN-AF Registry
Source: Glob Heart. 2020 Apr 10;15(1):32. doi: 10.5334/gh.767 (PMC7218765; doi:10.5334/gh.767)
Supplement: Supplementary material. — A full English version of the design of the Registry of Atrial Fibrillation and Embolic Risk in Mexico (CARMEN-AF), to assist our global audience to a better approaching to the original article published in Spanish in 2016 in Archivos de Cardiología de México. [file gh-15-1-767-s2.pdf]

Supplementary material

Diseño de un registro de fibrilacion auricular y riesgo embolico en Mexico: CARMEN-AF

(Fibrila**C**ion **A**uricular y **R**iesgo de Tro**M**boembolias **EN** Mexico)*

* This translation corresponds to the original article published in Spanish in:
J.A. González-Hermosillo, M.F. Márquez, S. Ocampo-Peña, R.Y. Villanueva, E. Velázquez, J.M. Enciso, R. Robledo, J. Gómez, S. Lara, Design of an atrial fibrillation and embolic risk registry in Mexico: CARMEN-AF, Arch. Cardiol. Mex. 87 (2017) 5–12. doi:10.1016/j.acmx.2016.11.003.

Resumen

Objetivo: La fibrilacion auricular (FA) es una de las arritmias mas comunes y su prevalencia aumenta con la edad. Se asocia con alto riesgo de embolia cerebral. La prevencion de dichas tromboembolias se realiza mediante anticoagulantes orales, que en nuestro pais parecen estar subutilizados. El Registro CARMEN-AF tiene como objetivo primario determinar cual es el estado actual de la tromboprofilaxis de la FA no-valvular en Mexico. Como objetivo secundario pretende conocer la morbi-mortalidad asociada a la FA no-valvular en por lo menos un año de seguimiento.

Metodos: El Registro CARMEN-AF es un estudio observacional, longitudinal, multicentrico y nacional, sobre el empleo de los anticoagulantes orales en pacientes con FA no-valvular, que pretende la inclusion de pacientes mayores de 18 años de edad diagnosticados con FA no-valvular durante los ultimos 6 meses y con al menos un factor de riesgo para desarrollar una tromboembolia de acuerdo con la escala de CHA2DS2-Vasc. Seran recolectados datos demograficos y clinicos en las visitas clinicas habituales a lo largo de un seguimiento de dos años. El reclutamiento inicio el 19 de septiembre de 2014 y se preve la inclusion del ultimo paciente el 18 de septiembre de 2016. Se estima la inclusion de 1,200 pacientes dada la incidencia de FA reportada a nivel mundial y tomando en consideracion la poblacion mexicana total.

Conclusiones: El registro de fibrilacion auricular y riesgo embolico en Mexico (CARMEN-AF) permitira conocer el estado actual de la tromboprofilaxis en pacientes con FA no-valvular, y

permitira obtener una panoramica del cumplimiento de las guias nacionales e internacionales de practica clinica en esta materia.

Conteo de palabras: 261

Palabras clave: Anticoagulante oral; Fibrilacion auricular; Registro; Tromboprofilaxis, Mexico

Design of an atrial fibrillation and embolic risk registry in Mexico: CARMEN-AF

Abstract

Objective: Atrial fibrillation (AF) is one of the most common arrhythmias, and its prevalence increases with age. It is associated with high risk of stroke. The prevention of such thromboembolism is accomplished with oral anticoagulants, which in our country seem to be underused. CARMEN-AF registry aims primarily to determine the current status of thromboprophylaxis of non-valvular AF in Mexico. A secondary objective is to know the morbidity and mortality associated with non-valvular AF in at least one year of follow-up.

Methods: CARMEN-AF registry is an observational, longitudinal, multicenter, and nationwide survey about the use of oral anticoagulants in patients with non-valvular AF. Patients 18 years old or older, diagnosed with AF during the last 6 months, and with at least one risk factor of thromboembolism based in the CHA2DS2-Vasc score are being selected. Demographic and clinical data will be collected during the visits to their usual clinic with a follow-up of two years. The recruitment began on September 19, 2014, and the inclusion of the last patient is expected on September 18, 2016. According to the reported incidence of AF globally and considering the total Mexican population, the inclusion of 1,200 patients is estimated.

Conclusions: The Atrial Fibrillation and Embolic Risk Registry (CARMEN-AF) will reveal the current status of thromboprophylaxis in patients with non-valvular AF and will allow to get an overview of the national and international clinical practice guidelines accomplishment in this area.

Word count: 236

Keywords: Atrial fibrillation; Oral anticoagulants; Registry; Thromboprophylaxis, Mexico

Introduction

Atrial fibrillation (AF) is the most common sustained arrhythmia encountered in clinical practice.[1,2] It affects about 1-2% of the general population[3], its prevalence increases with age, and it has multiple causes, among which are: heart failure, diabetes mellitus type 2, and hypertension[4]. AF is an important risk factor of stroke, as it is responsible for 25% of ischemic stroke, and 50% of cardioembolic stroke. Patients with AF have a cerebral ischemia incidence of 7% anually[5]. Besides, it is demonstrated that AF increases in-hospital mortality[6], increases the number of hospitalizations, and decreases quality of life[7]. Thus, AF represents a public health problem with an associated high economic cost[8,9].

One of the main objectives of AF treatment is the prevention of thromboembolism with oral anticoagulants (OAC). The AF therapeutic guidelines[5,10] provide recommendations about the suitability of a patient for OAC treatment according to different stroke risk stratification scores ($CHADS_2$ y CHA_2DS_2-VASc)[11] and bleeding risk (HAS-BLED)[12]. The Mexican Clinical Practice Guideline follows these same recommendations but does not emphasize the benefits of OAC in the more long-lived patients or with a high risk, which are usually treated with antiplatelet drugs[13].

Both direct OAC (DOAC), and vitamin K antagonists (VKA), have shown to be effective in preventing stroke in patients with AF[14-15]. However, adherence to vitamin K antagonists is highly variable, with a dropout rate in the first year of treatment that can reach up to 25%, and multiple drug interactions. Non-compliance with medication is associated with a poor perception by the patient about the need of taking medications or fear of adverse effects[16]. To improve the adherence more information is needed, so the patients be aware of the risk of their illness, consider it seriously, and get involved in essential prevention and treatment behaviors.

CARMEN-AF registry aims primarily to determine the current status of thromboprophylaxis of non-valvular AF in Mexico in order to identify the characteristics of the current use of OAC in Mexico. A secondary objective is to know the morbidity and mortality associated with this arrhythmia and the efficacy and safety in thromboprophylaxis of atrial fibrillation with the use of new and specific oral anticoagulants, in at least one year of follow-up.

Methods

The CARMEN-AF registry is an observational, longitudinal, multicenter, and nationwide survey about non-valvular AF. The CARMEN-AF registry is proposed as the first registry of the treatment of thromboembolic risk in non-valvular AF in Mexico that will have national representation. The participation of major health institutions, both public and private, of each state to ensure that the registry is representative of the country is planned. It seeks for wide geographical distribution to compare the quality of care between the different geographical areas of the country.

The follow-up period of each patient will be for a minimum of two years with periodic evaluations every three months. Data will be registered in an electronic case report form (Annex 2) stored in a centralized database in which all the necessary data for the study will be included. The study variables will be (1) demographic, (2) associated risk factors, (3) from diagnosis and characteristics of atrial fibrillation, as well as its clinical manifestations, (4) results of laboratory tests and cabinet examinations, and (v) clinical variables of important outcomes (morbidity-mortality). Data collection will be performed from the usual clinical practice through the participating researchers themselves and the patient's medical history at the time of inclusion in the study.

Inclusion and exclusion criteria

Candidates for the registry are those patients of any gender, older than 18 years who have documented by a 12-lead ECG, rhythm strip, Holter ECG monitoring or atrial electrograms of a pacemaker, at least one episode of AF in the last 6 months. All patients diagnosed with AF at the baseline visit must be eligible to receive antithrombotic prophylaxis with OAC and must have at least one risk factor for thromboembolism according to the CHA2DS2-VASc score. Female gender must be accompanied by some other risk factor, otherwise, it will not be coded for the risk score measured by CHA2DS2-VASc. Patients with AF of transient causes, AF onset in immediate postoperative or 3 months in cardiac surgery, terminal illness, mental inability to take anticoagulants, inability to fulfill the follow-up visits, already programmed for ablation of pulmonary veins, pregnant or lactating women, will be not eligible for inclusion in this registry (Table 1).

Data analysis

Descriptive and differential statistics will be used according to the observation obtained. For continuous variables, mean, standard deviation (SD), standard error of the mean (SEM), 95% confidence interval (CI 95%) minimum, percentile 25 (P25), median or percentile 50 (P50), percentile 75 (P75) and maximum, number of patients (n) and number of lost data will be obtained as descriptive statistics. For categorical variables, % will be obtained from the total and number of patients (n) in each category. Lost data will be treated as a new category.

Differences between continuous variables with normal distribution will be examined by Student's t-test. The Wilcoxon Sum-Rank Test will be used when continuous variables have failed in normality tests. To analyze categorical variables, chi-square will be used by the Fisher exact test or Yates correction. A two-tailed test with a value of $p < 0.05$ will be considered statistically significant. Logistic regression analysis will be used to select independent predictors in those variables in which a value of $p < 0.01$ has been obtained through univariate regression analysis. Kaplan-Meier survival curves and the Cox proportional hazard model will be used to adjust the survival analysis. A $p < 0.05$ will be considered statistically significant.

Results

The recruitment phase began on September 19, 2014 and will end on September 18, 2016. The participation of a total of 1,200 patients is estimated. A first preliminary analysis will be carried out with the first 1,000 patients. In September 2016, the cross-sectional analysis of all included patients will be performed (1,200 patients are calculated), and then one and two-year follow-up analysis will be performed.

The participating medical societies that support this Registry are, in alphabetical order: Asociacion Mexicana para la Prevencion de la Aterosclerosis y sus Complicaciones (AMPAC), Asociacion Nacional de Cardiologos al Servicio de los Trabajadores del Estado (ANCISSSTE), Asociacion Nacional de Cardiologos del Centro Medico La Raza A.C., Asociacion Nacional de Cardiologos de Mexico A.C. (ANCAM), Instituto Nacional de Cardiologia “Ignacio Chavez”

(INCICH), Sociedad Mexicana de Cardiologia A.C. (SMC), y la Sociedad Mexicana de Electrofisiologia y Estimulacion Cardiaca (SOMEEC).

Currently, there are 51 participating centers confirmed by the time being to include patients in the registry (Table 2).

Discussion

As in the rest of the world, in Latin America, the increase of life expectancy of population increases the incidence of ischemic stroke, and it is feared that its mortality will increase in the coming years[17,18]. Between 2003 and 2005 stroke occurred in 6.9% of the Mexican population, and it was the main cause of mortality in urban areas, affecting 14.9% of the mentioned population[19]. In 2010, 0.9% of the expenses of the Mexican public health sector were registries of acute stroke, of which 20,298 (43.9%) were ischemic[20]. On the other hand, the epidemiology of AF in Latin America is little known. In 2007, it was estimated that 60.7% of arrhythmias in Mexico were due to AF, of which 65.7% were non-valvular.

An important part of AF treatment is the prevention of thromboembolism. Anticoagulant therapy is highly effective in reducing the risk of stroke in these patients[21]; however, each patient must be assessed individually to determine the risks and benefits of antithrombotic therapy. Current clinical practice guidelines recommend administering anticoagulants according to the different risk stratification scores, in order to make treatment decisions according to the score obtained[22]. Thus, the Canadian guidelines advise the use of the $CHADS_2$ score to assess the risk of stroke, and the HAS-BLED score to assess the risk of bleeding, and the use of DOAC (dabigatran, rivaroxaban or apixaban) against VKA[23]. The USA guideline recommends the use of antithrombotic agents as a rule, except in contraindicated cases[24]. Finally, the European guidelines recommend the use of the CHA_2DS_2-VASc and the HAS-BLED scores to measure the risks of stroke and bleeding, respectively, and the use of DOAC against aspirin and VKA[10]. All these recommendations are based on the results obtained in phase III studies such as ARISTOTLE, RE-LY or ROCKET AF[25-28], which have shown advantages in the safety, efficacy,

and management of DOAC compared to VKA[29]. The recommendations of the Mexican clinical practice guideline also favor treatment with oral anticoagulants[5].

Despite the existing recommendations and the evidence in favor of treatments with OAC, it is estimated that only between 15% and 79% of patients receive this treatment properly[30]. VKA have complex pharmacokinetics that requires continuous monitoring of prothrombin time, frequent dose adjustments, with a high risk of bleeding, in addition to multiple interactions with other drugs and foods[30]. Ease of administration, low interaction with other drugs and reduced need for surveillance of DOAC, make an expected increase in the use of anticoagulant therapy in patients with AF[31].

Adherence to VKA is very variable. The ATRIA study documented a 25% dropout in the first year in patients treated with VKA[32], while the IN-RANGE study documented that 92% of patients missed a dose and 36% missed more than 20% of treatment[33]. This lack of adherence is one of the main causes of embolic events. On the other hand, there is no information on DOAC adherence, although it is expected to be greater than with VKA because of their fixed dosage, ease of administration, unnecessary monitoring, greater efficacy, and safety. To obtain a good follow-up in medication it is necessary to reduce the asymmetry of information, but it is also important to follow the recommendations of the international clinical practice guidelines.

Conclusion

The CARMEN-AF study will allow obtaining information about the current status of thromboprophylaxis in patients with non-valvular AF in Mexico. Anticoagulant treatment strategies in a total of 1,200 patients will be analyzed and their effectiveness will be evaluated. In addition, the degree of compliance with the clinical practice guidelines may be assessed, which will allow the development of an educational strategy to improve adherence to this type of treatment.

Ethical disclosures

Protection of human and animal subjects. The authors state that for this investigation no experiments have been performed on humans or animals.

Confidentiality of data. The authors state that they have followed the protocols of their work center on the publication of patient data.

Right to privacy and informed consent. The authors state that in this article there are no patient data.

Funding

Bayer, Boehringer-Ingelheim, and Pfizer unrestrictedly support the CARMEN-AF Registry, only for academic purposes.

Conflicts of interest

The Registry has unrestrictedly support from the Pharmaceutical Industry. They did not intervene in the development or the design of the CARMEN-AF Registry. Data collection will be performed by a specialized agency, independent of the sponsoring industry. Data analysis will be the responsibility of the Scientific Committee members who will be also in charge of coordinating the publications that may be generated from these analyses.

None of the authors involved in this research have a conflict of interest of any kind with Bayer, Boehringer-Ingelheim, Pfizer, Medicaweb or with any of the participating medical societies.

Acknowledgements

The authors would like to thank the Mexican Society of Cardiology for their endorsement, and MedicaWeb for the technical support provided in the development of this nationwide Registry.

References

1. Kakkar AK, Mueller I, Bassand JP, et al. International longitudinal registry of patients with atrial fibrillation at risk of stroke: Global Anticoagulant Registry in the FIELD (GARFIELD). Am Heart J. 2012; 163:13-9.

2. Iturralde-Torres P, Lara-Vaca S, Cordero-Cabra A, et al. Diseño de un registro multicentrico para evaluar control de ritmo contra control de la frecuencia en fibrilacion auricular: Registro Mexicano de Fibrilacion Auricular (ReMeFA). Arch Cardiol Mex 2011; 81:13-7.

3. Go AS, Hylek EM, Phillips KA, et al. Prevalence of diagnosed atrial fibrillation in adults: national implications for rhythm management and stroke prevention: the AnTicoagulation and Risk Factors in Atrial Fibrillation (ATRIA) Study. JAMA 2001; 285:2370-5.

4. Benjamin EJ, Levy D, Vaziri SM, et al. Independent risk factors for atrial fibrillation in a population-based cohort. The Framingham Heart Study. JAMA 1994; 271(11):840-4.

5. Castaño-Guerra RJ, Franco-Vergara BC, Baca-Lopez FM, et al. Guia de practica clinica. Diagnostico y tratamiento de la fibrilacion auricular. Rev Med Inst Mex Seguro Soc. 2012; 50:213-31.

6. Gonzalez-Pacheco H, Marquez MF, Arias-Mendoza A, et al. Clinical features and in-hospital mortality associated with different types of atrial fibrillation in patients with acute coronary syndrome with and without ST elevation. J Cardiol. 2015; 66:148-54.

7. Rivero-Ayerza M, Scholte op Reimer W, Theuns DAMJ, et al. New-onset atrial fibrillation is an independent predictor of in-hospital mortality in hospitalized heart failure patients: results of the EuroHeart Failure Survey. Eur Heart J 2008; 29:1618-24.

8. Go AS, Mozaffarian D, Roger VL, et al. Heart disease and stroke statistics- 2013 update: a report from the American Heart Association. Circulation 2013; 127:e6-e245.

9. Fuster V, Ryden LE, Cannom DS, et al. ACC/AHA/ESC 2006 guidelines for the management of patients with atrial fibrillation: full text: a report of the American College of Cardiology/American Heart Association Task Force on practice guidelines and the European Society of Cardiology Committee for Practice Guidelines (Writing Committee to Revise the 2001 guidelines for the management of patients with atrial fibrillation) developed in collaboration with

the European Heart Rhythm Association and the Heart Rhythm Society. Europace 2006; 8:651-745.

10. Camm J, Kirchhof P, Lip GYH, et al. Guidelines for the management of atrial fibrillation. Eur Heart J 2010; 31:2369–429.

11. Olesen JB, Torp-Pedersen C, Hansen ML, et al. The value of the CHA2DS2-VASc score for refining stroke risk stratification in patients with atrial fibrillation with a CHADS2 score 0–1: A nationwide cohort study. Thromb Haemost 2012; 107:1172–9.

12. Pisters R, Lane DA, Nieuwlaat R, et al. A novel user-friendly score (HAS-BLED) to assess 1-year risk of major bleeding in patients with atrial fibrillation: the Euro Heart Survey. Chest 2010; 138:1093-100.

13. Martinez-Flores JE. Reflexiones sobre la guia clinica en fibrilacion auricular. Rev Med Inst Mex Seguro Soc 2012; 50 (2): 117-22.

14. Hart RG, Pearce LA, Aguilar MI. Meta-analysis: antithrombotic therapy to prevent stroke in patients who have nonvalvular atrial fibrillation. Ann Intern Med 2007; 146(12):857-67.

15. Laupacis A, Dalen J, Feinberg W. Antithrombotic therapy in atrial fibrillation. Chest 1995; 108:352S-359S.

16. Kimmel SE, Chen Z, Price M, et al. The influence of patient adherence on anticoagulation control with warfarin. Results from the International Normalized Ratop Adherence and Genetics (IN-RANGE) study. Arch Intern Med 2007; 167:229-35.

17. Hart RG, Benavente O, McBride R, et al. Antithrombotic therapy to prevent stroke in patients with atrial fibrillation: a meta- analysis. Ann Intern Med 1999; 131:492-501.

18. Demographics and Retirement Policy in Brazil, Chile, and Mexico. Global Aging Initiative. Center for Strategic and International Studies. 2009. http://csis.org/files/media/csis/pubs/090324_gai_english.pdf Accessed March 21,2013.

19. Ferri CP, Acosta D, Guerra M, et al. Socioeconomic factors and all cause and cause-specific mortality among older people in Latin America, India, and China: A population-based cohort study. PLoS Med 2012; 9(2): e1001179. doi:10.1371/journal.pmed.1001179

20. Chiquete E, Ruiz-Sandoval JL, Murillo-Bonilla LM, et al. Egresos por enfermedad vascular cerebral aguda en instituciones publicas del sector salud de Mexico: Un analisis de 5.3 millones de hospitalizaciones en 201. Rev Mex Neuroci 2012; 13:252-8.

21. Lip GYH. Recommendations for thromboprophylaxis in the 2012 focused update of the ESC guidelines on atrial fibrillation: a commentary. J Thromb Haemost 2013; 11: 615-26.

22. Camm AJ, Lip GY, De Caterina R, et al. 2012 focused update of the ESC guidelines for the management of atrial fibrillation: an update of the 2010 ESC guidelines for the management of atrial fibrillation—developed with the special contribution of the European Heart Rhythm Association. Europace 2012; 14:1385-413.

23. Skanes AC, Healey JS, Cairns JA, et al. Focused 2012 update of the Canadian Cardiovascular Society atrial fibrillation guidelines: recommendations for stroke prevention and rate/rhythm control. Canadian J Cardiol 2012; 28:125-36.

24. Anderson JL, Halperin JL, Albert NM, et al. Management of patients with atrial fibrillation (compilation of 2006 ACCF/AHA/ESC and 2011 ACCF/AHA/HRS recommendations). A report of the American College of Cardiology/American Heart Association task force on practice guidelines. Circulation 2013; 127:1916-26.

25. Granger CB, Alexander JH, McMurray JJ, et al. Apixaban versus warfarin in patients with atrial fibrillation. N Engl J Med 2011; 365:981-92.

26. Connolly SJ, Ezekowitz MD, Yusuf S, et al. Dabigatran versus warfarin in patients with atrial fibrillation. N Engl J Med 2009; 361(12):1139-51.

27. Patel MR, Mahaffey KW, Garg J, et al. Rivaroxaban versus warfarin in nonvalvular atrial fibrillation. N Engl J Med 2011; 365(10):883-91.

28. Tendera M, Syzdoł M, Parma Z. ARISTOTLE RE-LYs on the ROCKET. What's new in stroke prevention in patients with atrial fibrillation? Cardiol J 2012; 19:4–10.

29. Buckingham TA, Hatala R. Anticoagulants for atrial fibrillation: why is the treatment rate so low? Clin Cardiol 2002; 25:447-54.

30. Michota F. Transitions of care in anticoagulated patients. J Multidiscip Healthc 2013; 6:215-28.

31. Singer DE, Chang Y, Fang MC, et al. The net clinical benefit of warfarin anticoagulation in atrial fibrillation. Ann Intern Med 2009; 151(5):297-305.
32. Ferguson C, Inglis SC, Newton PJ, et al. Atrial fibrillation and thromboprophylaxis in heart failure: the need for patient-centered approaches to address adherence. Vasc Health Risk Manag 2013; 9:3–11.
33. Horne R. Patients' beliefs about treatment: the hidden determinant of treatment outcome? J Psychosom Res 1999; 47:491-5.

Table 1. Inclusion and exclusion criteria.

Inclusion criteria

-Sex: Male or female.

-Age: ≥ 18 years old.

-Patient with diagnosis of chronic AF (permanent) documented by at least 2 electrocardiogram tracings taken on different days in the last 6 months. Tracings can be by 12-lead electrocardiogram, rhythm strip, or 24-hour Holter monitoring.

-Patient with diagnosis of paroxysmal/persistent AF documented for at least 30 seconds by 12-lead electrocardiogram, rhythm strip, 24-hour Holter monitoring, or atrial electrograms from a pacemaker. It is required that at least one episode has occurred and documented in the last 6 months.

-Patient with at least one risk factor for thromboembolism evaluated by CHA_2DS_2-VASC score.

Exclusion criteria

-AF of transient causes (thyrotoxicosis, alcohol intoxication, myocardial infarction in acute phase, pericarditis, myocarditis, electrocution, pulmonary embolism or other lung disease, electrolyte or metabolic disorder, etc.).

-AF onset in immediate postoperative or 3 months in cardiac surgery.

-Terminal illness.

-Mental inability to take anticoagulants.

-Inability to fulfill the follow-up visits.

-Patient already programmed for ablation of pulmonary veins.

-Pregnant or lactating women.

-Contraindication for oral anticoagulation (anemia, thrombocytopenia, severe liver or kidney damage, etc.).

Table 2. Participant centers

STATE	***CITY***	***CENTER***
**AGUASCALIENTES**	Aguascalientes	Cardiologica Aguascalientes
**BAJA CALIFORNIA**	Tijuana	Hospital Angeles de Tijuana
		Hospital Regional No. 1, IMSS
		ISSSTECALI
		Plaza Medical
**BAJA CALIFORNIA SUR**	La Paz	Hospital General La Paz, ISSSTE
**CAMPECHE**	Campeche	Hospital General de Especialidades INDESALUD
		Hospital General de Zona No.1, IMSS
**CHIAPAS**	Tuxtla Gutierrez	Policlinica de especialidades
**CHIHUAHUA**	Chihuahua	CIMA Chihuahua
**CIUDAD DE MEXICO**	Mexico City	Centro Medico Nacional "20 de noviembre", ISSSTE
		Centro Medico Nacional "La Raza", IMSS
		Hospital ABC
		Hospital Central de Alta Especialidad Norte, PEMEX
		Hospital de Cardiologia, Centro Medico Nacional "Siglo XXI", IMSS
		Hospital General de Mexico
		Instituto Nacional de Cardiologia "Ignacio Chavez"
		Instituto Nacional de Ciencias Medicas y Nutricion "Salvador Zubiran"
**COAHUILA**	Torreon	Unidad Medica de Alta Especialidad No. 71, IMSS
**COLIMA**	Colima	Centro Medico “Puerta de Hierro”
**DURANGO**	Durango	Hospital General de Durango
**ESTADO DE MEXICO**	Toluca	ISSEMYM Toluca
**GUANAJUATO**	Guanajuato	Unidad Medica de Alta Especialidad No. T-1, IMSS
	Leon	Hospital Angeles de Leon
**GUERRERO**	Acapulco	Hospital General de Acapulco
	Iguala	Hospital General de Iguala
**HIDALGO**	Pachuca	Consultorio privado, Dr. Lechuga
		Hospital General de Pachuca
**JALISCO**	Guadalajara	Centro Medico Nacional de Occidente, IMSS
		Hospital Civil de Guadalajara
**MICHOACAN**	Uruapan	Hospital General de Uruapan, SSA
**MORELOS**	Cuernavaca	Instituto Mexicano de Trasplantes
**NAYARIT**	Tepic	Consultorio privado, Dr. Varela
**NUEVO LEON**	Monterrey	Hospital San Jose
**OAXACA**	Oaxaca	Clinica Molina
**PUEBLA**	Puebla	Hospital Angeles de Puebla
		Hospital General del Sur de Puebla, SSA

**QUERETARO**	Queretaro	Instituto Corazon de Queretaro
**QUINTANA ROO**	Cancun	Hospital General de Zona No.3, IMSS
**SAN LUIS POTOSI**	San Luis Potosi	Hospital Angeles de San Luis Potosi
		Hospital Central "Morones Prieto"
**SINALOA**	Culiacan	Hospital Angeles de Culiacan
		Hospital Civil de Culiacan
**SONORA**	Obregon	Centro Medico Nacional del Noroeste, IMSS
**TABASCO**	Villahermosa	Hospital Regional de Alta Especialidad Villahermosa, SSA
**TAMAULIPAS**	Tampico	Hospital Angeles de Tampico
		Hospital Regional Tampico, PEMEX
**VERACRUZ**	Veracruz	Unidad Medica de Alta Especialidad No. 14, IMSS
**YUCATAN**	Merida	Star Medica Merida
**ZACATECAS**	Zacatecas	Hospital General No. 26, ISSSTE
		Hospital San Agustin

ANNEX 1

Study participants

Scientific Committee: Dr. J Antonio Gonzalez- Hermosillo (National Coordinator); Dr. Manlio F. Marquez Murillo (President); Dr. Salvador Ocampo Peña (Secretary); Dr. Guillermo Ceballos; Dr. Alejandro Cordero; Dr. Marcelo Jimenez; Dr. Armando Garcia; Dr. Efrain Gaxiola; Dr. Jorge Gomez; Dr. Enrique Martinez; Dr. Luis Molina; Dr. Gerardo Rodriguez-Diez; Dr. Humberto Rodriguez; Dr. Juan Pablo Benitez.

Financial Committee: Dr. Luis Molina; Dr. Bernardo Quintana; Dr. Ruben Yza Villanueva, AMPAC; Dr. Enrique Velazquez, ANCCMN “LA RAZA”; Dr. Jose Manuel Enciso, ANCAM; Dr. Rogelio Robledo, ANCISSSTE; Dr. Jorge Gomez (temporally), SMC; Dr. Susano Lara, SOMEEC.

Pharmaceutical Industry Representatives: Dra. Alejandra Meaney, Bayer; Dra. Yuriria Valle, Boehringer-Ingelheim; Dra. Ma. Eugenia Pastrana, Pfizer.

Contract Research Organization: Medicaweb, S. A. de C. V.

ANNEX 2

Case Report Form

Institucion: ____

Variables Demograficas

Iniciales:	Fecha de nacimiento: ___/___/______	Sexo: ( ) Masculino ( ) Femenino
Numero de Registro Institucional :		Fecha de Reclutamiento: (dia/mes/año) / /
Cobertura de salud: ( ) Privado ( ) Sector Salud ( ) IMSS ( ) ISSSTE ( ) PEMEX ( ) Otro		
Lugar de Residencia (Estado): Tipo de residencia: ( ) Urbana ( ) Rural		
Medico responsable (Especialidad): ( ) Electrofisiologo ( ) Cardiologo ( ) Neurologo ( ) Hematologo<br>( ) Medicina Interna ( ) Medico General ( ) Otro, especifique:		
Lugar de reclutamiento: ( ) Urgencias ( ) Hospitalizacion ( ) Consulta externa		Elegibilidad ( ) si ( ) no

Factores de Riesgo

Peso: Kg.		Estatura: m.		
( ) Hipertension Arterial	( ) Diabetes Mellitus Complicaciones ( ) Nefropatia ( ) Retinopatia ( ) Neuropatia			
( ) Distiroidismo Tipo: ( ) Hipertiroidismo ( ) Hipotiroidismo				
( ) Insuficiencia Cardiaca<br>Clase Funcional ( ) I ( ) II ( ) III ( ) IV		( ) Tumor Cardiaco	( ) Enfermedad Arterial Coronaria	
( ) Evento Cerebrovascular Previo ( ) Isquemico ( ) Hemorragico ( ) No determinado				
( ) Miocardiopatia<br>( )Hipertensiva ( )Isquemica ( )Restrictiva ( )Idiopatica		( ) Otras Miocardiopatias<br>(Especifique):		
( ) Ataque Isquemico Transitorio Previo	( ) Disfuncion del Nodo Sinusal			
( ) Enfermedad Arterial Periferica	( ) Cardiopatia Congenita ( ) Tipo, Especifique:			
( ) Enfermedad Aortica:<br>( ) Estenosis ( ) Insuficiencia ( ) DLAo		( ) Cirugia Cardiaca: ( ) Valvular ( ) Revascularizacion<br>( ) Otra		
( ) Enfermedad Carotidea Aterosclerosa		( ) Angioplastia coronaria previa		
( ) Enfermedad Tromboembolica Venosa ( ) Pericarditis				
Enfermedad pulmonar tipo ( ) EPOC ( )Asma ( ) SAHOS ( ) HAPulm				
( ) Insuficiencia Renal Cronica ( ) EI ( ) EII ( ) EIII ( ) EIV ( )EV		( ) Trombocitopenia	( ) Apnea Obstructiva del sueño	
( ) Enfermedad Acido Peptica ( ) Ulcera gastrica o duodenal ( ) Reflujo gastroesofagico ( ) ambos				
( ) Osteoporosis	( ) Enfermedad Hepatica Cronica	( ) Tabaquismo	( ) Anemia	( ) Alcoholismo
( ) Antecedentes de Hemorragia ( ) HTDA ( ) HTDB ( ) EVC Hemorragico ( ) Epistaxis<br>( ) Hemorragia conjuntival ( ) Equimosis ( ) Hematoma espontaneo ( ) Otros				

Variables Diagnosticas de Fibrilacion Auricular

Metodo diagnostico de fibrilacion auricular (seleccione uno) :<br>( ) Electrocardiograma de 12 derivaciones ( ) Tira de Ritmo<br>( ) Monitoreo electrocardiografico de 24 horas ( ) Monitorizacion de marcapaso permanente<br>Tipo: ( )Paroxistica ( )Persistente ( )Permanente

Manifestaciones Clinicas

( ) Dolor Toracico	( ) Palpitaciones	( ) Presincope
( ) Mareos	( ) Disnea	( ) Sincope
( ) Descompensacion de insuficiencia cardiaca	( ) Fatiga	( ) Edema
( ) Desencadenamiento de angina	( ) Asintomatico	

Electrocardiograma

Ritmo: ( ) FA ( ) Sinusal*	
Duracion QRS: __________ ms	Duracion onda P*: __________ ms
( ) Bloqueo de rama derecha	Intervalo PR*: __________ ms
( ) Bloqueo de rama izquierda	Intervalo QT*: __________ ms
Frecuencia cardiaca _______________	Frecuencia ventricular media*_____________

Ecocardiografia Basica

Auricula derecha : Dimensiones vista apical de 4 camaras (Fin de la sistole ventricular):<br>Longitudinal ________mm Transversal ______________mm	
Auricula izquierda: Dimensiones vista apical de 4 camaras (Fin de la sistole ventricular):<br>Longitudinal ________mm Transversal ______________mm	
( ) Ecogenicidad Espontanea ( ) Leve ( ) Moderada ( )grave	
Imagen ocupativa ( ) No ( ) Si ___________________	Sugerente de trombo ( )Si ( ) No Describir________________
( ) Aneurisma del Septum interauricular Desplazamiento ______mm	
Se realizo estudio contrastado (Solucion salina agitada) ( ) No ( ) Si<br>El estudio sugiere permeabilidad de foramen oval ( ) No ( ) Si	
( ) Comunicacion interauricular<br>(Este es un criterio de eliminacion, el paciente no puede ser incluido si la FA puede estar relacionada con una enfermedad valvular)	
Ventriculo derecho<br>Dilatado ( ) No ( ) Si Diametro antero-posterior (en diastole) _________ mm	
Ventriculo izquierdo<br>Dilatado ( ) No ( ) Si Diametro antero-posterior (en diastole) _________ mm<br>Grosor SIV __________mm Grosor PP___________mm Fraccion de expulsion ________%	
( ) Valvulopatia Mitral ( ) Funcional ( ) Organica ( ) Leve ( ) Moderada ( ) Severa	
( ) Valvulopatia Aortica ( ) Funcional ( ) Organica ( ) Leve ( ) Moderada ( ) Severa	
( ) Valvulopatia Tricuspidea ( ) Funcional ( ) Organica ( ) Leve ( ) Moderada ( ) Severa	

Anticoagulantes

Terapia de anticoagulacion oral: ( )No ( ) Si Tipo:<br>( ) Acenocumarina ( ) Warfarina Presentacion_________ Dosis X dia

L	M	M	J	V	S	D

Valor de INR Inicial: (*Solo aplica para pacientes con anticoagulantes tipo acenocumarina o warfarina)<br>--<br>( ) Dabigatran ( ) Rivaroxaban ( ) Apixaban Dosis: _________ Frecuencia : ( ) 1x1 ( ) 1x2
**Antiplaquetarios**
Uso actual de antiagregantes plaquetarios ( ) No ( ) Si<br>Acido acetilsalicilico ( ) dosis _____ mg/dia Clopidogrel ( ) dosis mg/dia ___________ Prasugrel ( ) dosis _____ mg/dia

Ticagrelor ( ) dosis mg/dia ___________ Otro antiplaquetario ____________________ dosis mg/dia ___________

Procedimientos Previos

( ) Cardioversion Electrica	Ritmo sinusal final: ( )No ( )Si
( ) Aislamiento de Venas Pulmonares	Ritmo sinusal final: ( )No ( )Si
( ) Cirugia de laberinto (maze)	Ritmo sinusal final: ( )No ( )Si
( ) Ablacion del nodo AV + Implante de Marcapaso	
( ) Implante de Marcapaso: Disfuncion del nodo sinusal ( ) Disfuncion del Nodo auriculo ventricular ( ) FA lenta ( )	

Pruebas de Laboratorio: Fecha: _____/____________/________

Hemoglobina g/dl	Acido urico mg/dl	Creatinina mg/dl	Bilirrubina total mg/dl
Hematocrito %	Colesterol mg/dl	Nitrogeno ureico (BUN) mg/dl	Bilirrubina directa mg/dl
Plaquetas /mm^3	Trigliceridos mg/dl	Glucosa en ayunas mg/dl	Bilirrubina indirecta mg/dl
Tiempo parcial de tromboplastina _______ seg<br>Testigo _____ seg	Peptido natriuretico tipo B	Alanina transaminasa (TGP)mg/dl	Gamma glutamiltransferasa mg/dl
Tiempo de trombina _seg<br>Testigo seg	Sodio mg/dl	Aspartato aminotransferasa (TGO) mg/dl	Fosfatasa alcalina mg/dl
	Potasio mEq/l	Proteinas totales g/dl	Calcio mg/dl
Leucocitos /uL	Magnesio mg/dl	Albumina g/dl	Cloro mg/dl

Llenado por: ____
